# Round the Hospitals

**Published:** 1916-04-08

**Authors:** 


### ROUND THE HOSPITALS.

Many hospital workers may be interested in an expert's description on page 40 of a useful flannel for bed-jackets, children's bed-gowns, shirts, and underwear, especially for the winter. A matron who has used it for some years speaks of it as being a very reliable flannel, though it is too heavy to be suitable for some purposes. The chief value of the Ruskin homespun lies in its wearing qualities, and other matrons may have discovered a similar fabric of lighter weight suitable for summer and babies' garments? Is this so?

A sister in charge of the laundry of a military hospital with 1,500 beds writes: "I am much interested to note that matrons of to-day are realising the importance of the laundry department. My experience is that hospital laundries are in many cases managed (or mismanaged) by incompetent people, generally a woman who can iron but has no knowledge of the all-important departments—*i.e.* the sorting-room and washhouse—result, great waste in time, labour, and materials, damage to goods, and unfair wear and tear of machines. Efficiently managed, there can be no doubt that the cost of running a hospital laundry fitted with the necessary machines is much less than sending the linen out; I endorse all your correspondents' views upon the other advantages, and, moreover, there is no risk of strong bleaches or injurious soap substitutes being used. A good working head with four maids, working full time, can do all the laundry work for a hospital having 200 in residence, patients and staff included."

Nursing in East Africa has suddenly assumed special interest owing to the advance of General Smuts' army against the German possessions there. Fifty nurses who are attached to the South African contingent under General Smuts have reached Nairobi, and are in residence at Muthaiga Club, the grounds of which have been transformed into a hospital camp for the sick and wounded. Muthaiga Club is situated four miles from Nairobi, amidst beautiful scenery. The club-house boasts electric light, a hot-water system, and other luxuries which are hardly to be expected in the heart of Africa, so that those of the wounded who are able to reach Nairobi should find themselves fortunate. It is probable, however, that the more serious cases in the recent fighting in the Kilimanyaro district will have been taken to Moschi, a German town recently occupied by General Smuts, where there is said to be a well-equipped hospital of 200 beds. Another contingent of fifty nurses from South Africa is at Zomba, in British Nyassaland. The difficulties attaching to the transit of the wounded may prove to be considerable.

The matron of the Crescent War Hospital, Croydon, is availing herself of the services of a lady who volunteered to visit the wards daily in the capacity of barber. The matron, Miss McCord, describes the experiment as most successful. The lady barber has now been at work for several months, and carries out her duties with unfailing punctuality to the entire satisfaction of the patients and ward sisters. This practical success in a new field of work for women is capable of development, and the lady barber may ultimately become an institution after the war, if the many women who might easily learn this art decide to render services to the wounded in this way.

A Matron writes: A word of praise is due to those trained nurses who are "carrying on" in women's special hospitals at home. In spite of great inducements, such as higher salaries and the excitement and glory of a new field of work, and in face of unworthy remarks such as "You still here! Why are you not nursing the wounded?" made by women, and perhaps the friends of their patients, yet they stick to their post. For they know that, if all trained nurses leave at once, these important women's hospitals would have to close down, as they are not training-schools, and the cases admitted cannot be nursed entirely by half-trained women. The matrons of these special hospitals have suffered, and are suffering, from the anxiety of losing their best nurses, with the uncertainty of filling the gaps by suitable women. This is the personal experience of a matron of a special hospital for women. What is the remedy?

The instinct possessed by dogs to recognise at once when life is present in a human body has proved the value of their use on the battlefield. Red Cross dogs are used by both the French and Russian Army Medical Departments, and are said to be doing excellent work. There are several hundred such dogs in use in the French Army, which are trained to search out the wounded and then to lead the ambulance men to them. It will be remembered that one of the sailors rescued from the *Formidable*, which was torpedoed off Lyme Regis, owes his life to a dog who drew attention to the body, which he refused to leave when the rescuers had passed the man as dead. Another instance of the same kind occurred in the retreat of the French before the advancing Germans at Fère Champenoise, when a dog was seen to have scratched a hole in a newly made grave and was dragging at a man's coat. Spades were brought, and the body of an unconscious but living man was recovered. The Germans claim to have saved over 4,000 lives by the use of dogs. Has any reader experience of the use of dogs by the British Army?

*** It is our invariable practice to pay for all items of news which we may publish. The minimum payment is 5s. Paragraphs of about twenty lines in length—that is to say, of 150 words or less—are preferred. In this section during the war we propose to give 10s. to the correspondent who may send us in any week the best incident connected with hospital life and work. In this way we hope to afford to all practical aid and encouragement; while those who reciprocate will be able to render us invaluable service. Contributions should be addressed to The Editor, The Hospital, 28 and 29 Southampton Street, Strand, London, W.C., and, to be in time for the current week's issue, should reach him by the first post on Mondays.

